# Multifunctional CD40L: pro- and anti-neoplastic activity

**DOI:** 10.1007/s13277-014-2407-x

**Published:** 2014-08-13

**Authors:** Aleksandra Korniluk, Halina Kemona, Violetta Dymicka-Piekarska

**Affiliations:** Department of Clinical Laboratory Diagnostics, Medical University of Bialystok, Bialystok, Poland

**Keywords:** CD40L, CD40, Inflammation, Apoptosis, Cancer

## Abstract

The CD40 ligand is a type I transmembrane protein that belongs to a tumor necrosis factor (TNF) superfamily. It is present not only on the surface of activated CD4+ T cells, B cells, blood platelets, monocytes, and natural killer (NK) cells but also on cancer cells. The receptor for ligand is constitutively expressed on cells, TNF family protein: CD40. The role of the CD40/CD40L pathway in the induction of body immunity, in inflammation, or in hemostasis has been well documented, whereas its involvement in neoplastic disease is still under investigation. CD40L ligand may potentiate apoptosis of tumor cells by activation of nuclear factor-κB (NF-κB), AP-1, CD95, or caspase-depended pathways and stimulate host immunity to defend against cancer. Although CD40L has a major contribution to anti-cancer activity, many reports point at its ambivalent nature. CD40L enhance release of strongly pro-angiogenic factor, vascular endothelial growth factor (VEGF), and activator of coagulation, TF, the level of which is correlated with tumor metastasis. CD40L involvement in the inhibition of tumor progression has led to the emergence of not only therapy using recombinant forms of the ligand and vaccines in the treatment of cancer but also therapy consisting of inhibiting platelets-main source of CD40L. This article is a review of studies on the ambivalent role of CD40L in neoplastic diseases.

## Introduction

The CD40 ligand (CD40L), also known as CD154, T-B activating molecule (TBAM), tumor necrosis factor (TNF)-related activation protein (TRAP), and gp39, is a type I transmembrane protein, a member of the TNF superfamily, together with lymphotoxin-α (LT-α, TNF-β), LT-β, FasL, CD30L, CD27L, and 4-1BBL 9 [1]. The CD40L gene is located on chromosome X (q26.3–q27.1) [2], contains five exons and four introns, and the protein that is produced (29 kDa) via translation is built up of 261 amino acids. The cell membrane presents the 32–33-kDA form, which indicates the posttranslation modification of the protein [3].

CD40L associated with cell membrane contains a globular TNF-like extracellular domain, long extracellular domain (ECD), short transmembrane domain (TMD), and a small cytoplasmic intracellular domain (ICD). [3] The extracellular structure of CD40L composed of beta sheet-alpha helix-beta sheet that is arranged in the so-called Greek key motif is specific to the TNF superfamily [4]. If CD40L is produced as a single polypeptide chain, it can be found on the cell surface as a homotrimeric complex. The long protein chain is accompanied by two shorter forms of CD40L, which through proteolytic hydrolysis are released from the cell membrane to the circulation, where they form soluble trimmers, sCD40L, having the receptor-binding ability [4, 5]. Since both the surface ligand and its soluble form have structural domains, they show high biological activity. Due to the presence of the KGD sequence (lysine-glycine-aspartic acid), they can bind to the GPIIb–IIIa receptor, and therefore, the ligand may play a role in platelet activation and stimulate further release of sCD40L [6, 7]. The trimeric protein structure facilitates signal transfer to the cell interior via binding to the receptor, whereas the TNF-like domain allows ligand binding to its main receptor-CD40. The presence of the ligand has been observed, first of all, on the surface of activated CD4+ T cells, but also on activated B cells and blood platelets, monocytes, natural killer (NK) cells, adipose cells, and basophils during inflammation [8]. It is assumed that over 95 % of CD40L originates from blood platelets [9]. Henn et al. [9] have shown that CD40L is stored in platelet granules α and released after their activation and degranulation.

The CD40 receptor is a transmembrane type I protein belonging to the TNF family, encoded by the gene located on chromosome 20 (q12–q13.2). It is present on the cell surface as a trimeric complex (40–45 kD) [10]. Structurally, CD40 contains a long ECD, transmembrane region, and short C-terminal cytoplasmic fragment [11]. Since the cytoplasmic part does not exhibit kinase activity, signals are transmitted mainly through the ligand-dependent recruitment of adaptor proteins of the TNF receptor-associated factor (TRAF) family [12]. The CD40 receptor has been found on the surface of B cells and on the membrane of the antigen-presenting cells (APCs), epithelial cells, endothelial cells, smooth muscle cells, fibroblasts, basophils, and blood platelets, where it is constitutively expressed [13, 14]. However, expression of the receptor can be induced by the combination of TNF-α with interferon (IFN)-γ. These molecules significantly increased de novo expression of CD40 on human endothelial and vascular smooth muscle cells, and this process is mediated by the simultaneous activation of nuclear factor-κB (NF-κB) by TNF-α and STAT-1α by IFN-γ [15, 16]. Activation of CD40 receptor by CD40 ligand on epithelial cells leads to the secretion of cytokines and chemokines by these cells, whereas on fibroblasts and endothelial cells, it contributes to their proliferation [17]. Interaction between CD40 and CD40L can be inhibited by specific antibodies like 4D11, a novel fully human anti-CD40 mAb which is produced by genetically modified mice. Also, lucatumumab and Chi 220 are anti-CD40 antibodies which inhibit ligation between receptor and ligand [18].

Neither CD40L nor its receptor CD40 can function independently, and only their interaction leads to the production of intracellular signal that is responsible for enhanced humoral and cellular response and for the cellular production of cytokines, chemokines, and adhesion proteins [19].

## CD40L in the immune response and inflammation

The first studies on the role of CD40L in the immune response were performed when the ligand was detected on the surface of T and B cells, confirming its involvement in cellular and humoral immune response. The activation of B cells by CD40L turned out to be essential in the process of lymphocyte proliferation, differentiation, and maturation and in isotope switching [10]. The interaction of CD40L with CD40 exerts an effect on the production of cytokines (IL-6, IL-10, TNF-α, LT-α), expression of adhesion molecules and co-stimulatory receptors (intercellular adhesion molecule (ICAM), CD23, B7.1/CD80, B7.2/CD86), as well as class I MHC, class II MHC, and transporter associated with antigen processing (TAP) present on B cells [20].

The significant role of the CD40L-CD40 system in the immune response was confirmed with the discovery of CD40L gene mutation, leading to the hyper-IgM syndrome (HIGM). In these patients, overproduction of IgM antibodies and lack of IgG, IgA, and IgE antibodies can be observed [3].

The binding of CD40L to CD40 present on endothelial cells and fibroblasts increases the secretion of metalloproteinases and activates the production of chemokines (IL-8, MCP-1, macrophage inflammatory protein (MIP)-1α, RANTES) and cytokines (IL-1, IL-6, IL-12, and TNF-α), which attract lymphocytes to the site of inflammation and increase expression of adhesion molecules (ICAM-1, VCAM-1, and E-selectin), responsible for the recruitment of monocytes and lymphocytes, thus leading to their accumulation in the internal vascular membrane [21].

Interaction between ligand and receptor causes overexpression of cycloxygenase 2 (COX-2) and prostaglandin E2 (PGE2) [22]. The soluble form of CD40L is able to activate blood platelets and stimulate the release of β-thromboglobulin (β-TG) and 5-hydroxytryptoamine from their granules, whereas cell membrane CD40L causes the release of regulated on activation, normal T cell expressed and secreted (RANTES), the protein of strong pro-inflammatory properties, from the platelets [23, 24].

The evidence for the involvement of the CD40/CD40L pathway in inflammation includes highly elevated expression of these molecules in inflammatory conditions of the colon, such as Lesniewski-Crohn disease or ulcerative colitis [19]. The pro-inflammatory and pro-thrombotic activities of the CD40/CD40L system have been also shown in diabetes [25], atherosclerosis [26], and cardiovascular diseases [27]. High level of sCD40L can be a prognostic risk factor of death, heart infarct, and recurrent angina in acute coronary syndromes and future cardiovascular episodes in healthy women [28]. It is also a reliable marker for the population of patients with acute coronary syndrome at a high risk of heart incidents [29].

## CD40L in neoplastic disease

The role of the CD40/CD40L pathway in the induction of body immunity, in inflammation, or in hemostasis has been well documented, whereas its involvement in neoplastic disease is still controversial.

**Fig. 1:**
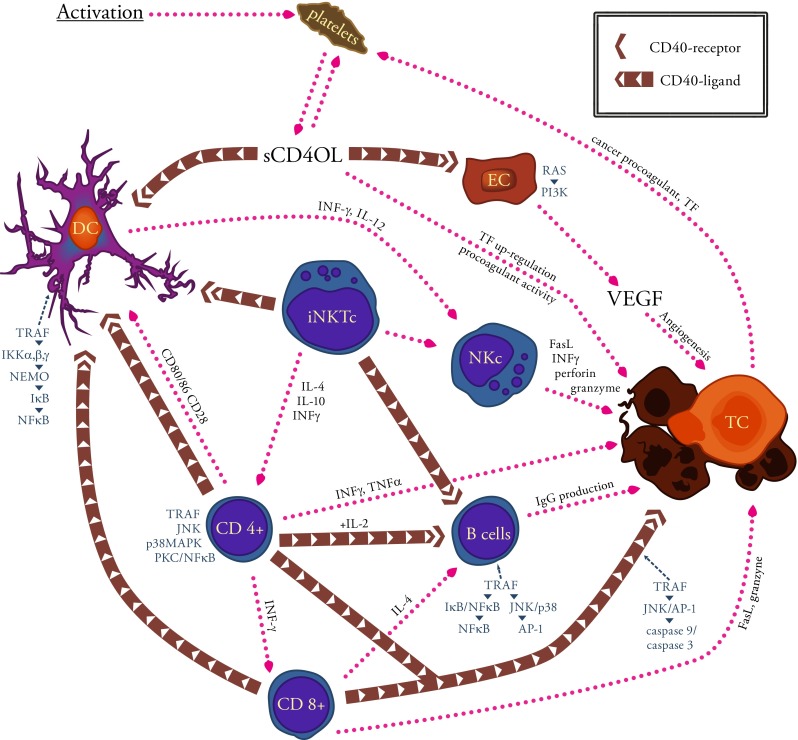
This figure illustrates some of the mechanisms by which CD40L can influence on different cell functions and the processes by which these altered cells can impact on host immunity in neoplastic diseases. CD40L is present as a membrane-bound form (mCD40L), present first of all on T lymphocytes (*CD4+*, *CD8+*, *iNKTc*, and *NKc*), and as a soluble form (sCD40L) derived primarily from active platelets. Interaction of the ligand with the receptor (CD40) may not only potentiate the anti-tumor immunity but also promote the development of cancer. The binding of CD40L with a receptor on dendritic cells (*DC*) leads to the expression of co-stimulatory molecules necessary for the correct antigen presentation and protects *DC* against apoptosis induced by factors derived from tumor cells (*TC*). Moreover, CD40L/CD40 interaction enhances the proliferation and maturation of lymphocytes B (*B cells*) and the production of antibodies against the tumor. mCD40L present on various cells induces apoptosis of tumor cells via the *TRAF*-*JNK*/*AP-1*-*caspase-9*/*caspase-3* pathway. On the other hand, the activation of endothelial cells (*EC*) may result in enhance *TF* pro-coagulant activity and high expression of VEGF, main mediator of tumor angiogenesis

Studies on CD40L have revealed that it enhances anti-neoplastic immune response of the body, inhibits tumor growth, and induces apoptosis of cancer cells [30, 31]. Subsequent reports have suggested that in many cancers, CD40 activation by its ligand results in a completely reverse situation, i.e., enhancement of tumor growth and progression (Fig. 1) [32, 33]. The effect induced by CD40L has appeared to depend not only on the type of cells that show the receptor expression but also on the strength of the signal transmitted by the ligand. High signal (the cell has many CD40 molecules) indicates apoptosis of cancer cells, whereas low signal (a small number of receptors) CD40L promotes cancer growth [34].

## CD40L and cancer progression

CD40L has a major contribution to immunological activity; however, many reports point at its ambivalent nature in neoplastic diseases. On one hand, the ligand activates the immune system to combat the cancer, but on the other, it stimulates tumor progression, growth, and metastasis formation.

### Induction of angiogenesis by CD40L induces VEGF production

Tumor growth and metastasis depend on angiogenesis and lymphangiogenesis caused by different factors released from host and tumor cells [35]. Binding of CD40L to CD40 present on endothelial cells leads to the expression of strongly pro-angiogenic factors, such as vascular endothelial growth factor (VEGF) or fibroblast growth factor 2 (FGF-2) that can promote in vivo angiogenesis. [36–38]. VEGF participates in mobilization of endothelial stem cells, which take part in formation of new blood vessels in tumor microenvironment [35]. In addition, VEGF increases expression of tissue factor (TF), which also promotes blood coagulation. Moreover, VEGF induces monocyte chemotaxis and activation and also impairs the immune system functions through the inhibition of dendritic cell maturation [39]. In gastric cancer, ligation of CD40 by CD40L causes up-expression of VEGF by PI3K pathway [40]. CD40L/CD40 interaction also has been shown to induce COX-2 expression in cells and subsequent VEGF production, what was confirmed by Miura et al. [41]. Tai YT et al. [42] suggested that in human multiple myeloma (MM) cells, CD40 activation by CD40L can induce secretion of VEGF by p53 pathway. In this case, VEGF stimulates IL-6 secretion in bone marrow stromal cells and thereby augments paracrine IL-6-mediated MM cell growth. What is more, Farahani et al. [43] shows that in cells from patients with chronic lymphocytic leukemia (CLL), CD40L also can up-regulate production of VEGF, which leads to CLL cell survival. This process depends, in part, on NF-κB activation. CD40L-induced survival of malignant cells depends on combined signaling by CD40 and VEGF receptor (VEGFR). Inhibition of CD40L-induced production of VEGF and cytokines (IL-6) and activation of signaling pathways, proliferation, and survival of CLL [44] and MM [42] cells is caused by lucatumumab (HCD122). HCD122 binds to CD40 and blockade CD40/CD40L interactions that induce apoptosis and mediate antibody-depended cellular toxicity on lucatumumab-bound CD40-expressed malignant cells. This antibody is currently in phase I/II clinical trials in CLL. VEGF can also enhance cleavage of membrane-bound CD40L and cause increased level of sCD40L. Inhibition of this process is possible through the use of bevacizumab-anti-VEGF antibody [18].

### Hematological malignancy

The activation of CD40 contributes to the increased survival and resistance to chemotherapy of follicular lymphoma, hairy cell leukemia, and CLL cells [45–47]. It is suggested that the co-stimulation of IL-4 and CD40L causes long-term proliferation of B cells and short-term proliferation and increased percentage of coat cells in hairy cell leukemia [48]. Kato et al. [49] showed that such interaction in diffuse non-Hodgkin’s lymphoma depending on the type of cancer cell line enhanced short- or long-term proliferation of cancer cells. Research conducted on the established cell lines (GDLBGCB-1 and GDLBGCB-2) and cells taken from the patients has proved that lack of IL-4 or introduction of antibodies against CD40L causes inhibition of cell proliferation. In some types of CLL, cell proliferation does not depend on endogenous CD40L, but on the stimulation with the ligand from tumor microenvironment. Pham et al. [50] suggest that endogenous CD40L which is present on aggressive B cells of lymphoma binds to CD40 and the signal transferred into the cell activates the NF-κB pathway.

The effect of CD40L on the action of many drugs has been reported. The CD40L induces resistance to a number of anti-cancer drugs, including doxorubicin or vinblastin. In non-Hodgkin’s lymphoma, lack of reaction to drugs is caused by caspase-independent and independent pathways [51], whereas in breast cancer, only by the caspase-dependent route [52]. A study conducted on transgenic mice showed that the administration of clopidogrel, a drug inhibiting platelet aggregation and CD40L release, decreased tumor size [53]. This may suggest that tumor growth inhibition by the drug is associated with an indirect decrease in CD40L expression [51]. The sCD40L/CD40 pathway activation in gastric cancer inhibited Fas- and drug-dependent apoptosis and also increased the motility of CD40-positive cancer cells, thus facilitating the formation of metastases, what was confirmed in humans [54]. Rui Li et al. [55], who investigated the expression of CD40 and CD40L on gastric cancer cells from patients, suggest that a simultaneous expression of the two molecules and permanent activation of the CD40/CD40L pathway have a major significance in neoplastic progression. CD40 activation inhibits apoptosis and promotes spread of transformed cells. Likewise, in cancer of the urinary bladder, the activity of the CD40/CD40L system protects cells against apoptosis and increases their survival, which is associated with the inhibition of CD95-dependent apoptosis [56]. The assessment of CD40 expression on the surface of pancreatic cancer cells revealed that at stage I of the disease, the cells were CD40-negative, whereas in patients with lymph node involvement and distant metastases, the cells exhibited high expression of CD40. Also, the serum level of sCD40L in these patients was significantly elevated. The two molecules correlated with cancer stage, which may indicate their involvement in the disease development. The use of shrCD40L on pancreatic cancer cell lines significantly inhibited their proliferation and enhanced apoptosis, although the effect was most pronounced in the cells showing high CD40 expression [57].

### Solid tumors

Rosseli et al. [58] showed a high level of sCD40L in patients with adenocarcinoma and squamous cell carcinoma of the lungs, with the highest level of the ligand observed in patients with distant metastases of the latter. Moreover, sCD40L showed a positive correlation with the pro-thrombotic system components, i.e., fragments of pro-thrombin 1 and 2 (F1 + 2), and the thrombin/anti-thrombin complex (TATc). The researchers forwarded the hypothesis that the pro-thrombotic agents released from lung cancer cells stimulate platelet activation and release of sCD40L, which in turn interacts with other types of CD40-positive cells, including tumor-associated macrophage (TAM) cells and cancer cells of the lungs. Also, Amirkhosravi et al. [59] suggested that the interaction of CD40L present on blood platelets with CD40 on tumor cells may potentially promote activation of coagulation on human melanoma cells A375 through the effect of increased expression of TF.

## Anti-neoplastic stimulation of immune response 

Research on CD40L indicates that the inhibition of cancer progression can be associated with the activation of two mechanisms by the ligand, namely, stimulation of anti-neoplastic immune response and induction of apoptosis of transformed cells.

The enhancement of immune response is inextricably associated with the activation of antigen-presenting cells (APCs), especially dendritic cells, monocytes, and B cells, showing CD40 expression. Cancer cells release a number of cytokines and soluble agents that have an immunosuppressive effect and cause APC dysfunction [60]. Most data seem to confirm a beneficial effect of CD40L/CD40 interaction on the function of dendritic cells (DC). It has been proved that CD40L/CD40 binding protects DC against apoptosis, by inducing the expression of the anti-apoptotic molecules Bcl-2 or serpin serine protease inhibitor 6 (SPI-6) [61, 62]. The application of recombinant CD154 therapy leads to the formation of mature dendritic cells, increased expression of adhesion and co-stimulatory molecules (ICAM-1, CD83, CD80/86), and secretion of pro-inflammatory cytokines (TNF-α, IL-6, IL-12) and chemokines (IL-8, MIP-1α), which confirms an exceptionally important role of the ligand in the activation of these cells [63]. CD40L, together with pro-inflammatory cytokines and INF-γ, is indispensable for the presentation of foreign antigens absorbed by dendritic cells (cross-priming) to T cells. It has been suggested that IL-12 and INF-γ regulate the expression of CD154 on effector cells, whereas the elevated level of the ligand may modulate NK cell cytotoxicity [64]. The ligand binding to the receptor present on monocytes and macrophages is associated with the activation of numerous immune processes involving these cells [65].

The activation of CD40 by CD40L enhances the expression of TLR-9 on macrophages, whereas its stimulation by sCD40L increases the activity of monocytes, especially in cancer of the uterine cervix, which is related to the activation of the NF-κB and MARK pathways [66]. Studies on the stimulation of CD40 present on cancer cell lines in the bladder, pancreas, or breast through the recombinant CD40L have shown increased expression of ICAM and Fas by cancer cells and the production of IL-6, IL-8, GROα, GM-CSF, and TNF-α. The incubation of transformed cells with the expression of CD40 and CD40L has been found to considerably inhibit their proliferation, disturbances in the cell cycle, and decreased cell viability [66]. Moreover, the administration of anti-CD40 antibodies has been observed to activate macrophages, thus enabling them to inhibit proliferation of melanoma B-16 cells and to stimulate them to interferon production [67]. CD40 binding to CD40L not only leads to the increased production of cytokines by immune cells, but also stimulates many types of cancers to release pro-inflammatory proteins. Cells of Hodgkin’s lymphoma due to CD40 activation release IL-8, IL-6, or TNF, which in consequence can increase T cell-dependent anti-neoplastic immune response [68]. Also, in acute lymphoblastic leukemia, the activation of CD40 present on cancer cells by CD40L enhances the secretion of MDC and TARC; i.e., the proteins that play a role of chemoattractans for CCR4+ T cells [69]. These lymphocytes possess CCR4 receptor on such chemokines as MPC-1, MIP-1, or RANTES. The presence of this receptor also allows migration of lymphocytes to the skin. High percentage of CCR4+ T cells have been observed in malignant skin lymphomas, such as mycosis fungoides or Sezary’s syndrome [70].

## CD40L activation-dependent apoptosis of cancer cells

Apart from its effect on the body immunity, the CD40L/CD40 system can inhibit cancer growth via enhanced apoptosis of cancer cells. Interestingly, binding of CD40L by CD40 induces the apoptosis of cancer cells, but not of the healthy ones. CD40L prevents death of fibroblasts, dendritic cells, monocytes or B cells, and enhances the death of transformed mesenchymal cells, epithelial cells, neurons, or hepatocytes. This phenomenon is referred to as “activation-induced cell death” (AICD) [31, 71, 72]. The ligand induces the apoptosis of CD40-positive cancer cells. The presence of CD40 molecule was first identified on cancer cells of urinary bladder. Subsequent research showed CD40 expression on cancer cells of the breast [73], ovary, intestines [74], liver [75], glioma [17], nasopharynx [76], melanoma [77], or lymphoma.

As shown by literature data, cells with CD40 on their surface very seldom express CD40L. Moreover, various cancer cell lines exhibit a varied level of CD40, which is associated with cancer stage and usually decreases with disease progression [30]. The receptor present on the transformed cells can bind to the cell membrane-bound ligand (mCD40L), soluble CD40L (sCD40L), recombinant CS40L (srhCD40L), and anti-CD40 antibody. The effect induced by the ligand-receptor interaction depends on the type of cells on which the molecules are located and on the form of the ligand that binds to the receptor.

The CD40L/CD40 pathway may activate CD95-dependent apoptosis, which has been proved in the research on neuroblastoma, and via the activation of caspase-8 that is induced by recombinant CD40L administration [78]. Investigations of normal and transformed epithelial cells of the urinary tract indicate that the activation of cancer cells by mCD40L, but not by sCD40L, leads to the growth and stabilization of TRAF-3 and causes activation of caspase-9 and caspase-3. In healthy cells, CD40L does not induce apoptosis but leads to a decrease in the activity of TRAF-3 and TRAF-2 [79]. The TRAF-2 factor exerts a positive effect on differentiation of B cells, whereas TRAF-3 inhibits the growth and blockade of NF-κB factor in cancer cells [79]. Other authors have investigated the significance of CD40 expression on cancer cells of the urinary tract. The incubation with CD40L leads to the apoptosis of transformed cells, whereas decreased expression of CD40 on these cells is associated with enhanced cancer progression [80].

It has been shown that CD40L may enhance apoptosis of solid tumor cells and transformed cells of blood cancers [17, 79].

### Hematological malignancy

The CD40/CD40L interaction on human and murine B cell lymphoma resulted in the arrest of cell cycle, which had a major significance for the induction and maintenance of tumor quiescent state. Also, in the case of Burkitt lymphoma, CD40 activation caused tumor growth inhibition, increased activity of Fas, and enhanced apoptosis of tumor cells [46]. Tong et al. [31] have demonstrated the expression of CD40 by MM cells and blockage of their progression by the use of srCD40L therapy. In turn, Young et al. [81] have suggested that the loss of CD154 expression on the myeloma cells leads to the ligand-dependent loss of epithelial growth regulation and uncontrolled inflammations and infections, which may contribute to the increased and uncontrolled susceptibility of patients to the development of cancer. It has been also observed that despite preserved expression of CD40 by oral basal epithelial cells, the loss of CD154 expression may contribute to epithelial metastasis [76]. Also, in patients with CD40L gene mutation (HIMN), an increase was noted in the incidence of cancer of the liver, pancreas, or bile ducts, which seems to confirm the Young et al. theory [82].

### Solid tumors

It is assumed that the level and type of the cytokines secreted by cancer cells depend on whether the ligand is membrane-bound or soluble. Numerous studies have shown that binding of both mCD40L and sCD40L to the receptor CD40 leads to the activation of NF-κB, although in the case of the transcription factor AP-1, the pro-apoptotic cascade is induced only by mCD40L [74]. The activation of NF-κB factor stimulates the release of IL-8, whereas AP-1 enhances the production of GM-CSF [75].

The results obtained by Georgopoulos et al. [74] indicate that the interaction between mCD40L and CD40 on colon cancer cells leads to the formation of a strong pro-apoptotic signal. Interestingly, the authors have revealed that this effect is induced only by mCD40L binding. Apoptosis of colon cancer cells was not observed when they were CD40-negative and subject to the action of sCD40L [79, 80]. mCD40L interaction with CD40-positive cancer cells was accompanied by increased production of IL-8 by transformed cells. It has also been shown that in the course of colon cancer, mCD40L affects the expression of GM-CSF. Growth inhibition or death in carcinoma cells mediated by CD40 can be accompanied also by induction of IL-6 production [74]. These cytokines have an ambivalent character. On one hand, up-regulated production of cytokines in response to CD40 activation can enhance the immunogenicity through augmented presentation of tumor-associated antigens by the tumors and increasing recruitment of immune cells into tumor sites, which was observed after CD40L stimulation [83]. Enhance production of IL-6, IL-8, TNF-α, and GM-CSF and corresponding caspase activation can contribute to CD40-dependent tumor growth inhibition [31]. On the other hand, prolonged production of IL-6 and GM-CSF and activation NF-κB pathway can exacerbate the inflammation and induce cancer progression through intensifying angiogenesis and inhibition of transformed cells apoptosis.

CD40L inhibits in vitro melanoma cell proliferation by inducing their apoptosis or stimulating the production of cytokines that activate the immune system (IL-6, IL-8, TNF-α) by cancer cells [77]. In another study, two independent teams assessed the role of the ligand and CD40 receptor activation in breast cancer. The results reported by Hirano et al. [84] suggest that the action of the CD40/CD40L pathway may inhibit the growth and enhancement of apoptosis of transformed cells. The use of genetically modified CD40L-srhCD40L by these authors caused death of cancer cells via Fas activation. In turn, Tong et al. [73] have revealed that breast cancer cell apoptosis does not require the presence of INF-γ, which was earlier suggested by Wingett et al. [85]. The authors showed that modified sCD40L by binding on cancer cells inhibits tumor growth, whereas the receptor-free transformed cells do not undergo apoptosis.

Some authors believe that the CD40L/CD40 pathway may inhibit cancer progression through only one mechanism, i.e., enhanced immune response or apoptosis induction. However, most researchers favor the theory that anti-neoplastic activity is only possible when both mechanisms by CD40L are involved in anti-cancer therapy.

Since CD40L and CD40 molecules are strong immunostimulators, they are considered useful in anti-cancer therapy. Studies on novel methods of treatment have been conducted on murine models, using anti-CD40 and anti-CD40L antibodies, recombinant CD40L, and transformed viral vectors, carrying encoding sequences/ligand genes or vaccines, to the production of which dendritic cells containing tumor peptides and CD40L gene are used [86, 87].

The activation of antigen-presenting cells (APCs, i.e., CD4+ T cells and dendritic cells) by CD40L present on CD4+T cells enhances the specific anti-cancer immune response. Therefore, the major therapeutic purpose was target activation of CD40, which leads to the intensified CD-dependent T cell activation and the resulting effective anti-cancer immune response. This assumption is accomplished with the use of various methods, including agonistic antibodies and recombinant forms of sCD40L. Currently, researchers are widely interested in the transfer of the CD40L encoding sequences to various cells, including cancer cells, fibroblasts, or dendritic cells. The introduction of the CD40L encoding sequence by means of viral vector (adenoviruses, retroviruses) to cancer cells leads to the enhancement of the ligand expression and increased activity of dendritic cells that stay in contact with cancer cells, which accelerates their maturation and induces anti-cancer activity [88].

One of the cancer treatment strategies is to use CD40-ligand/interleukin-2 vaccines. Chronic lymphocytic leukemia (B-CLL) cells express many of tumor-associated and tumor-specific antigens but lack co-stimulatory molecules, which are required for effective antigen presentation. Up-regulating of CD80, CD86, and CD54 can be caused by CD40L interaction with CD40, and further immunostimulatory effect can be enhance by IL-2 [89]. Biagi et al. [90] prepared autologous B-CLL cells expressing human CD40 ligand (hCD40L) and human interleukin-2 (hIL-2). Vaccine leads to CD4+ and CD8+ effector cells reactive with autologous B-CLL cells and consequently to immunomodulatory effect. In non-Hodgkin’s lymphoma, cells after cultured with embryonic lung fibroblasts were transducted by adenoviral vector with Adh CD40L and Adh IL2. This combination caused enhanced initial T cell activation and generation autologous T cells against B-NHL cells [91].

Murine model research on urinary bladder cancer has shown that gene therapy with the use of vectors that carry the CD40L encoding sequence (AdCD40L) has high potential in the inhibition of tumor growth. The introduction of AdCD40L has led to enhanced expression of IL-12 and generation of specific all-systemic defense response against tumor. At the same time, the development and function of regulator T cells were suppressed in the lymph nodes [92]. Kipps et al. [93] proposed gene therapy for patients suffering from chronic lymphoblastic leukemia. Leukemia cells were transduced by Ad-CD154, which promoted antigen presentation and generation of autologic T cell response. This caused the formation of cytotoxic T cells, specific to leukemia cells [65]. Similar results have been reported by researchers who studied the effect of gene therapy with the use of CD40L on gastric cancers [94], non-small-cell lung cancer [95], breast cancer [85], cancers of the uterine cervix, and the prostate [96]. Studies on the new more effective and safer forms of vectors that carry genes into the cells are still performed. For instance, conditionally replicative oncolytic adenovirus (AdEHCD40L) containing hybrid promoter ERE/HRE that regulates transgenic gene CD40L has been proposed for the treatment of breast cancer. The application of gene therapy has led to the inhibition of tumor growth, enhanced apoptosis of cancer cells, and turned out to be completely safe for the body [97, 98]. Vardouli et al. [99] assessed the effect of therapy using recombinant non-replicating adenovirus, showing the expression of CD40L, replication-defective recombinant adenovirus (RAd)-hCD40L (RAd vector expressing hCD40L) on cancer cells. Their findings indicate that permanent activation of CD40 by CD40L inhibits proliferation and enhances apoptosis of cancer cells. Transduction of Rad-hCD40L to CD40-positive cancer cells of the urinary bladder, uterine cervix, and ovary inhibits strong proliferation of these cells [99].

CD40L genes can be transduced not only to cancer cells, but also to the healthy ones in order to enhance their activity. Kikuchi et al. [100] have forwarded the hypothesis that genetically modified CD40L-positive dendritic cells will be able to activate each other and enhance anti-cancer response after being introduced to tumor microenvironment. Research in vivo has confirmed the theory. The use of modified dendritic cells (AdmCD40L-modified syngenic DCs) increased the immune response and suppressed tumor growth. In order to increase the activity of antigen-presenting cells, therapy is used with anti-CD40 antibodies (SGN-40). In MM, the use of these antibodies significantly enhanced cell apoptosis and decreased the expression of the receptor for IL-6 [101].

CD40L on cell membrane is known as a homotrimeric molecule, which allows its binding to the receptor and activation of the signaling path. At present, an increasing number of literature data suggest that such a structure is indispensable for the ligand functioning, although the transmitted signal is too weak to exert the maximum effect [102]. The modified multimeric forms of CD40L considerably more strongly activate the receptor than the homotrimeric form does [103]. This correlation was applied to CD40L therapies. Naito et al. [108] have suggested an innovative preparation of human recombinant CD40L (CD40L Tri), in which the ECD of CD40L is connected to the “trimeric motif” of CD40L (CD40L-Tri) by a long elastic peptide bond. CD40L-Tri considerably increases the population of CD19 B cells and induces their transition into antigen-presenting cells. The B cells activated by CD40L-Tri can effectively stimulate the T cell-dependent response. Also, the multimeric molecule, SP-D-CD154, being the fusion of CD40L and lung surfactant protein D (SP-D), activates proliferation of B cells more strongly than the CD40L-Tri does [104].

## Conclusions

The knowledge of the role of CD40L in normal functioning of the immune system has initiated a number of studies that have revealed not only its involvement in the functioning of T and B cells, but also its key role in the pathogenesis of such diseases as atherosclerosis, cardiovascular disorders, and cancer. The latest research on the ligand has concentrated around its practical therapeutic use. At present, the recombinant ligand is effectively used in the treatment of renal failure and has very promising implications in the therapy for breast cancer and pancreatic cancer. Moreover, new forms of the modified ligand, vaccines that contain its gene, and multimetric forms of the protein are produced. In a few years, therapies that use CD40L are likely to be part of combined therapy.

## Abbreviations

TNF: Tumor necrosis factor
TRAF: TNF receptor-associated factor
TAP: Transporter associated with antigen processing
RANTES: Regulated on activation, normal T cell expressed and secreted
VEGF: Vascular endothelial growth factor
FGF-2: Fibroblast growth factor 2
TF: Tissue factor
APCs: Antigen-presenting cells
DC: Dendritic cells
NK cell: Natural killer cells
GM-CSF: Granulocyte-macrophage colony-stimulating factor
CLL: Chronic lymphocytic leukemia
MM: Multiple myeloma
